# NELFE-Dependent MYC Signature Identifies a Unique Cancer Subtype in Hepatocellular Carcinoma

**DOI:** 10.1038/s41598-019-39727-9

**Published:** 2019-03-04

**Authors:** Hien Dang, Yotsawat Pomyen, Sean P. Martin, Dana A. Dominguez, Sun Young Yim, Ju-Seog Lee, Anuradha Budhu, Ashesh P. Shah, Adam S. Bodzin, Xin Wei Wang

**Affiliations:** 1 Laboratory of Human Carcinogenesis, Center for Cancer Research, National Cancer Institute, Bethesda, Maryland United States; 2 Department of Systems Biology, Division of Cancer Medicine, UT MDACC, Houston, TX United States; 3 Translational Research Unit, Chulabhorn Research Institute, Bangkok, 10210 Thailand; 4 Department of Surgery, Division of Surgical Research, Thomas Jefferson University, Philadelphia, PA United States; 5 Department of Surgery, Division of Transplantation, Thomas Jefferson University, Philadelphia, PA United States

## Abstract

The MYC oncogene is dysregulated in approximately 30% of liver cancer. In an effort to exploit MYC as a therapeutic target, including in hepatocellular carcinoma (HCC), strategies have been developed on the basis of MYC amplification or gene translocation. Due to the failure of these strategies to provide accurate diagnostics and prognostic value, we have developed a Negative Elongation Factor E (NELFE)-Dependent MYC Target (NDMT) gene signature. This signature, which consists of genes regulated by MYC and NELFE, an RNA binding protein that enhances MYC-induced hepatocarcinogenesis, is predictive of NELFE/MYC-driven tumors that would otherwise not be identified by gene amplification or translocation alone. We demonstrate the utility of the NDMT gene signature to predict a unique subtype of HCC, which is associated with a poor prognosis in three independent cohorts encompassing diverse etiologies, demographics, and viral status. The application of gene signatures, such as the NDMT signature, offers patients access to personalized risk assessments, which may be utilized to direct future care.

## Introduction

Hepatocarcinogenesis is a complex process associated with numerous changes at both the genetic and epigenetic levels. The activation of oncogenes and dysregulation of signal transduction pathways, such as Negative Elongation Factor E (NELFE)/MYC, Wnt/β-catenin, hepatocyte growth factor (HGF)/c-Met, and transforming growth factor β (TGFβ) all contribute to hepatocarcinogenesis^1^. One common change found in many cancers, including hepatocellular carcinoma (HCC), is the alteration of the MYC oncogene^2^. MYC regulates more than 15% of the transcriptome, controlling cellular processes such as proliferation, differentiation, apoptosis and metabolic programming^2^. MYC amplification is the most common alteration in cancers and is often used as a biomarker. Additionally, dysregulated MYC signaling without gene amplification, mutation or translocation is also observed^3^. The complex nature of MYC alteration is a potential rationale as to why some MYC targeted therapies fail^4^. Since MYC and its network are altered by complex mechanisms, characterization of the MYC gene copy number or translocation alone is not sufficient to identify MYC-driven tumors. The present study seeks to evaluate a gene signature to predict MYC-driven tumors in HCC.

We have previously demonstrated that the activation of NELFE enhances MYC-induced hepatocarcinogenesis by supporting the tumor transcriptome^5^. Our findings suggest NELFE promotes hepatocarcinogenesis by either regulating the stability of downstream MYC targets or by directly interacting with the MYC protein to enhance transcription. Furthermore, we identified a subset of oncogenic MYC targets regulated by NELFE, called NELFE-dependent MYC targets (NDMTs), in HCC tumor tissue and have functionally validated these findings through *in vitro* studies. While the NELFE/MYC axis may be a potential therapeutic target, there are currently no NELFE directed therapies. Historically, MYC has been considered an undruggable target due to its complex role in the cell^6^. Despite this preconception, attempts to target MYC have been made by identifying tumors in which MYC is overexpressed^6,7^. This approach, however, does not address tumors that may be driven by NELFE/MYC dysregulation whereby MYC overexpression is not required to drive MYC-induced tumorigenesis^2,5,6^.

HCC is the second most common cause of cancer related deaths worldwide^8^. The incidence of HCC continues to rise in the United States, where the overwhelming majority of patients are diagnosed with advanced disease. As such, most patients are deemed non-surgical candidates, eliminating curative therapeutic options. Despite considerable efforts toward improving diagnosis, progress in durable treatment options have remained elusive, most offering minimal improvement in survival. Due to these challenges, HCC remains among the most difficult to treat malignancies with a 5-year survival of less than 15% in the United States^8^. The array of underlying liver diseases associated with HCC, heterogeneity of the tumor, advanced disease at presentation, ineffective chemotherapy, and high recurrence rates all contribute to the overall poor prognosis of HCC^8,9^. With the current paradigm offering patients very little, a strategy to stratify HCC subtypes according to their tumor biology is needed to improve therapeutic response. In the current study, we establish an NDMT gene signature composed of 20 genes regulated by NELFE/MYC with the goal of identifying a unique subtype associated with poor survival in HCC.

## Results

### Establishment of the NELFE Dependent MYC Target (NDMT) gene signature

**Figure 1:**
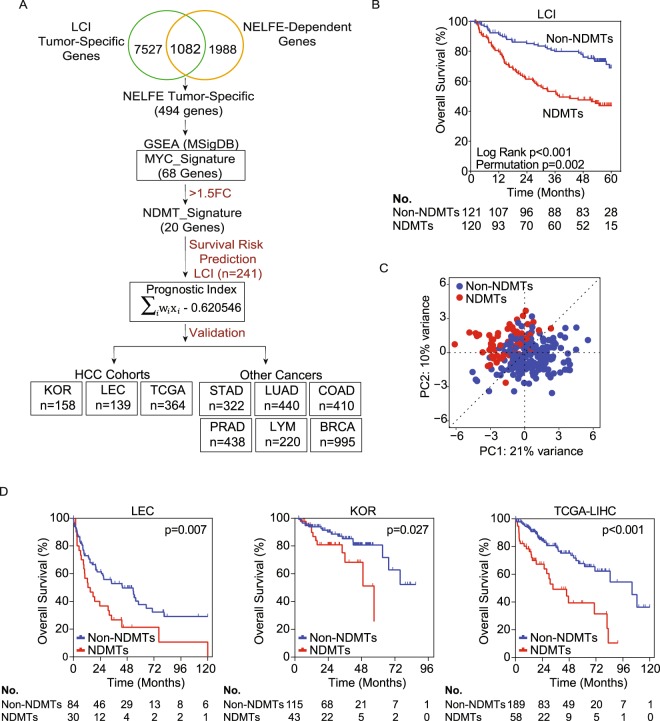
Development of the 20-NELFE Dependent MYC Target (20-NDMT) signature. (**A**) The work flow of the study from establishing the NDMT signature to validation in HCC cohorts and other tumor types. (**B**) Survival risk prediction analysis using the Liver Cancer Initiative (LCI) cohort. P values displayed are calculated by Mantel-Cox log-rank test and permutation test with 10-fold cross validation. (**C**) Principal component analysis (PCA) of the LCI cohort using only the 20 genes from the signature (PC: principal component). (**D**) Kaplan-Meier analysis of three independent HCC cohorts. P-values are from Mantel-Cox log-rank test in the Laboratory of Experimental Carcinogenesis (LEC), Korean (KOR) cohort, and The Cancer Genome Atlas-Liver Hepatocellular Carcinoma (TCGA-LIHC).

We previously demonstrated that the oncogenic RNA binding protein (RBP) NELFE supports the tumor transcriptome by regulating MYC and its targets^5^. Furthermore, we identified 68 oncogenic NELFE/MYC target genes associated with poor survival and demonstrated that in HCC, patients with elevated NELFE gene expression have a poor prognosis^5^. To further establish a robust gene signature, we filtered genes with least 1.5-fold change (tumor vs. non-tumor) from the 68-gene list, which resulted in 20 genes (Fig. 1A, Supplementary Fig. S1). To establish the signature’s prognostic index, we performed survival risk prediction analysis using the Liver Cancer Institute (LCI) cohort (see Methods) (GSE14520)^10,11^. Briefly, the algorithm uses all 20 genes and fits them into a Cox proportional hazards model to provide an assessment of whether the association of each gene expression to survival data is statistically significant^12^. Accordingly, the NDMT gene signature stratified patients with a significant difference in overall survival (OS) (Fig. 1B). The subgroup of patients with attenuated survival is referred to as NDMTs, while those with favorable survival are henceforth referred to as Non-NDMTs. The NDMT subtype had a median survival of only 37.9 months compared to the Non-NDMT subtype, which did not yet reach the median survival by the end of data collection (Log rank p < 0.0001). The molecular signature’s cross-validated misclassification rate was significantly lower than by random chance (permutation p = 0.002) (Fig. 1B). In addition, principal components analysis using the NDMT gene expression in the LCI cohort further confirmed the existence of two distinct tumor types (Fig. 1C).

We next performed a multivariate Cox proportional hazards regression analysis (Table 1) comprised of univariate variables with a p < 0.05, which included the NDMT signature status, stage, cirrhosis status and alpha-feto protein (AFP) levels of >400 ng/ml. Both tumor size and microvascular invasion, which were significant in the univariate analysis, were excluded due to collinearity (Table 1). The NDMT prognostic signature was an independent predictor of OS with a hazard ratio (HR) of 1.8 (95% CI = 1.1–2.9, p = 0.010). In addition, we ran our analysis for both the Barcelona-Clinic Liver Cancer (BCLC) and TNM Classification of Malignant Tumors (TNM) staging, which were found to be significant (TNM: HR = 1.8, 95% CI = 1.8–4.8 p < 0.001, BCLC: HR = 3.0, 95% CI 1.9–4.7, p < 0.001). Neither AFP nor cirrhosis status were found to be significant.

**Table 1:** Univariate and multivariate Cox regression analyses of the LCI cohort (n = 241).

Clinical variable	Hazard Ratio (95% CI)	p value^a^
**Univariate Analysis**		
20-NDMT Signature (NDMTs vs. Non-NDMTs)	2.3 (1.5–3.5)	**<0.001**
Sex (Male vs. Female)	1.8 (0.9–3.7)	0.111
Age, y (≥50 vs <50)	0.8 (0.5–1.2)	0.268
Cirrhosis (Yes vs No)	4.8 (1.2–20.2)	**0.025**
BMI (≥24 vs <18.5)	0.7 (0.3–2.1)	0.565
HBV status (AVR-CC vs CC)	1.4 (0.9–2.2)	0.153
Child-Pugh class (B vs A)	1.4 (0.8–2.5)	0.247
ALT (≥50 vs <50)	1.2 (0.8–1.8)	0.370
AFP (>400 ng/ml vs ≤400 ng/ml)	1.7 (1.1–2.6)	**0.009**
Tumor size (>3 cm vs ≤3 cm)	2.5 (1.5–4.3)	**<0.001**
Histological grade (II-IV vs. I)	0.9 (0.4–2.2)	0.914
Multinodular tumor (Yes vs No)	1.6 (1.0–2.4)	0.052
Microvascular invasion (Yes vs No)	1.7 (1.1–2.6)	**0.009**
BCLC staging (B&C vs A)	3.7 (2.4–5.8)	**<0.001**
TNM staging (II + III vs I)	2.9 (1.8–4.8)	**<0.001**
**Multivariate Analysis** ^**b**^		
20-NDMT Signature (NDMTs vs. Non-NDMTs)	1.8 (1.1–2.9)	**0.010**
Cirrhosis (Yes vs No)	3.4(0.8–13.9)	0.091
AFP (>400 ng/ml vs ≤400 ng/ml)	1.2 (0.8–2.1)	0.367
BCLC staging (B&C vs A)	3.0 (1.9–4.7)	**<0.001**
Multivariate Analysis^c^		
20-NDMT Signature (NDMTs vs. Non-NDMTs)	1.9 (1.2–3.0)	**0.010**
Cirrhosis (Yes vs No)	3.0 (0.7–12.5)	0.211
AFP ( > 400 ng/ml vs ≤ 400 ng/ml)	1.4 (0.9–2.2)	0.581
TNM staging (II + III vs I)	2.4 (1.4–3.9)	**0.001**

Note: Bold indicates significant p values.

Abbreviations: AVR-CC, active viral replication chronic carrier; CC, chronic carrier; AFP-alpha-fetoprotein; ALT, alanine aminotransferase; BCLC, Barcelona clinic liver cancer; BMI, body mass index; NA, not available.

^a^Univariate analysis.

^b^Multivariate analysis, Cox proportional hazards regression adjusting for Cirrhosis, AFP status, and BCLC staging.

^c^Multivariate analysis, Cox proportional hazards regression adjusting for Cirrhosis, AFP status, and TNM staging.

### Validation of the NDMT signature in other HCC cohorts

Next, we tested the gene signature in three independent HCC cohorts (Supplementary Table 1). Accordingly, the Laboratory of Experimental Carcinogenesis (LEC) cohort (GSE1898 and GSE4024) (n = 139) consists of patients of European background who are HBV/HCV positive, the Korean (KOR) cohort (GSE15765) (n = 158) consists mostly of HBV positive patients, and The Cancer Genome Atlas-Liver Hepatocellular Carcinoma (TCGA-LIHC) cohort (n = 364) consists of Asian, African, Hispanic and Caucasian patients who are HBV/HCV positive^13–16^. Kaplan-Meier log rank analyses were performed to assess the relationship between the NDMT subtype and OS. In the LEC cohort, the Non-NDMT subtype experienced a median survival of 43.8 months compared to 14.0 months in the NDMT subtype (p = 0.007). In the TCGA-LIHC cohort, the median survival of Non-NDMTs were 104.2 months compared to 33.5 months in NDMTs (p < 0.001). Finally, in the Korean cohort, the NDMT subtype’s median survival was 57 months, whereas at the end of data collection, the Non-NDMT subtype had yet to be defined (p = 0.027). Of note, the median OS in the LEC cohort is significantly shorter than the LCI, TCGA-LIHC or the Korean cohort, which is consistent with the late stage disease observed at diagnosis. Together, these data suggest the gene signature is a predictor of poor survival and is robust in predicting the NDMT subtype among different HCC cohorts across various races/ethnicities and mixed etiology.

To test whether the gene signature is independent of other prognostic factors, including sex, age, cirrhosis status, AFP, TNM staging, BCLC staging, and BMI in the validation cohorts, we performed Cox regression analysis in the LEC, TCGA-LIHC and the Korean datasets (Table 2). Univariate Cox regression analyses revealed the NDMT signature was a significant predictor of survival in the TCGA-LIHC (HR = 2.9, 95% CI = 1.8–4.8, p < 0.001), LEC (HR = 1.9, 95% CI = 1.1–3.1, p = 0.015), and Korean (HR = 2.3, 95% CI = 1.1–5.1, p = 0.035) cohorts. In the TCGA-LIHC cohort, TNM stage (HR = 2.1, 95% CI = 1.3–3.6, p = 0.005) and BCLC staging (HR = 3.1, 95% CI = 1.5–6.3, p = 0.002) were also significant predictors of survival (Table 2). In addition to the NDMT signature, microvascular invasion status (HR = 3.2, 95% CI = 1.5–6.9, p = 0.003) and TNM staging (HR = 2.2, 95% CI = 1.0–4.8, p = 0.046) were also predictive of OS in the Korean cohort. The NDMT signature was the only significant predictor of survival in the LEC and thus, no further multivariate analysis was performed (Table 2).

**Table 2:** Univariate and multivariate Cox regression analyses of the TCGA-LIHC, LEC, and KOREAN cohorts.

Clinical variable	HR (95% CI)	p value^a^	HR (95% CI)	p value^a^	HR (95% CI)	p value^a^
Univariate Analysis	TCGA-LIHC		LEC		KOREAN	
20-NDMT Signature (NDMTs vs. Non-NDMTs)	2.9 (1.8–4.8)	**<0.001**	1.9 (1.1–3.1)	**0.015**	2.3 (1.1–5.1)	0.035
Sex (Male vs. Female)	0.7 (0.5–1.2)	0.225	1.4 (0.8–2.3)	0.227	1.1 (0.4–2.9)	0.828
Age, years (≥50 vs <50)	1.2 (0.6–2.2)	0.582	0.7 (0.4–1.1)	0.148	0.9 (0.4–2.0)	0.726
Cirrhosis (Yes vs No)	0.8 (0.4–1.7)	0.535	1.4 (0.9–2.2)	0.191	2.3 (0.8–6.4)	0.128
AFP (>400 ng/ml vs ≤400 ng/ml)	1.1 (0.6–2.0)	0.836	1.4 (0.9–2.4)	0.161	1.3 (0.6–2.9)	0.501
Microvascular invasion (Yes vs No)	1.3 (0.8–2.2)	0.334	1.4 (0.6–3.3)	0.398	3.2 (1.5–6.9)	**0.003**
TNM staging (II + III vs I)	2.1 (1.3–3.6)	**0.005**	NA	NA	2.2 (1.0–4.8)	**0.046**
BCLC Staging (B&C vs A)	3.1 (1.5–6.3)	**0.002**	0.7 (0.2–2.9)	0.595	2.0 (0.7–5.5)	0.167
Tumor size (>3 cm vs ≤3 cm)	NA	NA	1.5 (0.7–3.2)	0.329	1.8 (0.7–4.7)	0.233
Histological grade (II-IV vs I)	1.1 (0.5–2.3)	0.790	0.5 (0.1–2.0)	0.322	NA	NA
Child-Pugh class (B vs A)	2.1 (0.9–5.1)	0.101	NA	NA	NA	NA
**Multivariate Analysis** ^**b**^						
20-NDMT Signature (NDMTs vs. Non-NDMTs)	2.6 (1.3–5.1)	**0.005**				
BCLC staging (B&C vs A)	3.1 (1.5–6.3)	**0.002**				
**Multivariate Analysis** ^**c**^						
20-NDMT Signature (NDMTs vs. Non-NDMTs)	2.6 (1.5–4.5)	**p < 0.001**				
TNM staging (II + III vs I)	2.1 (1.2–3.5)	**0.006**				

Note: Bold indicates significant p values.

Abbreviations: AFP, alpha-fetoprotein; ALT, alanine aminotransferase; BCLC, Barcelona clinic liver cancer; BMI, body mass index; NA, not available.

^a^Univariate analyses.

^b^Multivariate analysis, Cox proportional hazards regression for Cirrhosis, AFP status, and BCLC staging.

^c^Multivariate analysis, Cox proportional hazards regression adjusting for Cirrhosis, AFP status, and TNM staging.

We next performed multivariate Cox regression analysis between the NDMT signature and significant predictors from the univariate analyses to investigate their relationship. In the TCGA-LIHC dataset, the NDMT signature remained an independent predictor of survival (HR = 2.6, 95% CI = 1.3–5.1, p = 0.005) along with BCLC staging (HR = 3.1, 95% CI = 1.5–6.3, p = 0.002) (Table 2). In addition, we also investigated TNM staging independently from BCLC as both staging systems consist of similar parameters, including tumor size and lymph node invasion. We found that the 20-NDMT signature (HR = 2.6, 95% CI = 1.5–4.5, p < 0.001) remained independent when using TNM staging (HR = 2.1, 95% CI = 1.2–3.5, p = 0.006) (Table 2), indicating its prognostic value. Notably, the number of available data points in the LEC and Korean datasets for the analyzed clinical factors in the NDMT subtype are small, which may not be adequate to draw any conclusions (See Supplementary Table 1).

### Performance of the NDMT signature

**Figure 2:**
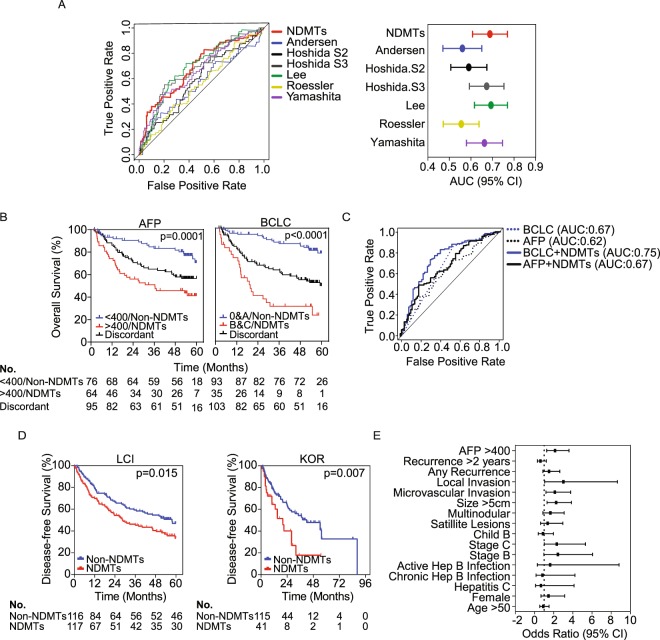
The NDMT subtype is associated with agressive tumors. (**A**) (Left) Receiver operating curve (ROC) analyses of different gene signatures compared to the NDMT signature in the LCI cohort at two-year time-points. (Right) Forest plot of the area under the curve (AUC) with 95% confidence interval. (**B**) Kaplan-Meier curve survival analyses of the LCI cohort stratified by AFP and BCLC staging with NDMT or Non-NDMTs. P value is from Mantel-Cox log rank analyses. (**C**) Time-dependent ROC curve analyses at two-years for Barcelona-Clinic Liver Cancer (BCLC), alpha-feto protein (AFP), BCLC + NDMT, or AFP + NDMT with associated AUCs. (**D**) Disease-free Kaplan-Meier survival analysis of the LCI and Korean cohort. (**E**) Odds ratio (±95% confidence interval) of logistic regression analyses of the LCI cohort.

To determine the signature’s potential use in the clinical setting, we performed time-dependent receiver operating characteristic (ROC) curve analyses on three of the four HCC cohorts with a cut-off at 2-years based on the finding that the 5-year survival rate is less than 15%. We elected to not analyze the Korean cohort due to lack of sufficient gene expression data for all samples. In addition, we compared our NDMT gene signature to six other known gene signatures including the Andersen signature^17^, Roessler Metastasis signature^10^, Hoshida signature^18,19^, Lee signature^13^, and Yamashita’s EpCAM signature^20^. In the LCI cohort, ROC analyses indicated the gene signature had the best predictive accuracy (area under the curve (AUC) = 0.69) compared to other published gene signatures (Fig. 2A). In the TCGA-LIHC and the LEC cohort, ROC curve analyses indicated the NDMT gene signature performed as well as other signatures and demonstrated acceptable predictive accuracy (AUC = 0.62 for TCGA-LIHC and AUC = 0.62 for LEC) (Supplementary Fig. S2A,B). However, the NDMT signature was outperformed by the Hoshida S3 signature in the TCGA-LIHC (AUC = 0.68) cohort and the Lee signature (AUC of 0.75) in the LEC cohort (Supplementary Fig. S2B). Together, these findings indicate that the NDMT signature identifies a very specific HCC subtype with NELFE/MYC signaling and its performance is stable across unique etiologies.

Clinical decisions are often guided by AFP and BCLC staging, both of which were independent predictors of OS in the LCI and TCGA-LIHC cohorts. Thus, we tested whether AFP levels or BCLC staging could improve the prognostic prediction of the NDMT signature. For AFP, we divided patients into subgroups based on a cutoff of 400 ng/ml, resulting in three groups: >400/NDMTs, <400/Non-NDMTs, or Discordant (patients with no correlation). Kaplan-Meier curve analyses in the LCI cohort showed that patients in the Non-NDMT subtype with AFP levels of <400 ng/ml had a significantly better OS than patients in the NDMT subtype with AFP levels >400 ng/ml (p = 0.0001). This finding was also observed in the TCGA-LIHC cohort (Fig. 2B (left), Supplementary Fig. S2C). Patients with AFP levels >400 ng/ml in the NDMT HCC subgroup had a median survival of 36.4 months in the LCI cohort and 33.5 months for TCGA-LIHC, whereas at the end of data collection, patients in the Non-NDMT subgroup with AFP levels <400 ng/ml or the Discordant group had yet to be defined in the LCI cohort (Supplementary Fig. S2C, left). In the TCGA-LIHC cohort, patients with AFP levels <400 ng/ml in the Non-NDMTs group had a median survival of 104.2 months and the Discordant group had a median of 45.7 months (Supplementary Fig. S2C, right). For BCLC staging, we stratified patients with BCLC stage 0 and A into A&0, BCLC stage B and C into B&C groups, which resulted into three distinct groups: A&0/Non-NDMTs, B&C/NDMTs, or Discordant. Survival analyses in both cohorts showed that NDMT patients with BCLC stages B&C had a worse OS compared to Non-NDMT patients with BCLC A&0 or the Discordant (p < 0.0001) group with a median survival of 19.2 months and 33.5 months for the LCI and TCGA-LIHC, respectively (Fig. 2B, Supplementary Fig. S2C, right). At the end of data collection, the median survival for the Non-NDMT subtype with BCLC staging A&0 had yet to be defined for both cohorts, whereas the median survival for NDMTs with BCLC staging B&C had a median survival of 59.2 months and 84.7 months for LCI and TCGA-LIHC, respectively. Together, these data indicate that patients in the NDMT subtype are likely to have high levels of AFP and BCLC staging B or C and only BCLC staging status may improve the NDMT’s survival predictive accuracy.

To test whether AFP levels or BCLC staging improves the OS predictive accuracy of the NDMT signature, we performed two-year dependent ROC curve analyses for both cohorts. We found that the NMDT signature had no effect on AFP for both the LCI (AUC of AFP = 0.62, AUC of AFP + NDMTs = 0.67) and TCGA-LIHC cohorts (AUC of AFP = 0.55, AUC of AFP + NDMTs = 0.54) (Fig. 2C, Supplementary Fig. 2C). However, in the LCI cohort, the NDMT signature improved OS prediction for patients with BCLC B&C stages (AUC of BCLC = 0.67, AUC or BCLC + NDMTs = 0.75) (Fig. 2C). This finding was not observed in the TCGA-LIHC cohort (AUC of BCLC = 0.55, AUC of BCLC + NDMTs = 0.55), which may be due to the smaller sample size (Supplementary Fig. 2C). These data indicate that the NDMTs subtype is more likely to consist of HCC patients with advanced disease, i.e. patients with BCLC staging B or C.

### NDMTs are associated with aggressive tumor types

We next assessed the gene signature’s predictive ability on disease-free survival (DFS) by performing log-rank analyses in two HCC cohorts, the LCI and Korean cohorts. Log-rank analyses revealed the NDMT subtype had an earlier time to progression than the Non-NDMT subtype in the LCI (p = 0.015) and Korean (p = 0.007) cohorts (Fig. 2D). While the NDMT subtype in the LCI and Korean cohort had a median recurrence of 19.1 and 20 months, the Non-NDMT subtype had a median recurrence of 36.9 and 54.6 months, respectively. Univariate Cox proportional hazards analyses revealed the NDMT signature (HR = 1.5, 95% CI = 1.1–2.1, p = 0.020), cirrhosis status (HR = 2.7, 95% CI = 1.3–5.7, p = 0.011), microinvasion (HR = 1.4, 95% CI = 1.0–1.9, p = 0.040) and BCLC stage (HR = 2.3, 95% CI = 1.6–3.3, p < 0.001) were also predictive of DFS. Consistent with previous work, multivariate Cox regression analyses revealed that BCLC stage (HR = 2.7, 95% CI = 1.6–4.4, p < 0.001) and cirrhosis status (HR = 2.6, 95% CI = 1.1–5.9, p = 0.024) remained independent predictors of DFS. The NDMT signature (HR = 2.3, 95% CI = 1.1–5.1, p = 0.035), microinvasion (HR = 3.2, 95% CI = 1.5–6.9, p = 0.003) and TNM stage (HR = 2.2, 95% CI = 1.0–4.8, p = 0.046) were also predictive of DFS. However, when we performed multivariate Cox regression analyses using only significant variables found in the univariate analyses, we observed that the 20-NDMT gene signature was no longer an independent predictor of DFS in both cohorts.

In addition to DFS and OS, the NDMT signature was evaluated as a predictor of aggressive tumor features. Using patient data from the LCI cohort, a variety of clinical factors were evaluated, including age, sex, viral status, stage at presentation, local tumor features and preoperative AFP. Univariate analysis showed that NDMT signature was associated with poor prognostic features such as size >5 cm (odds ratio (OR) = 2.26, 95% CI = 1.31–3.77), microvascular invasion (OR = 2.12, 95% CI = 1.19–3.77), and local invasion into surrounding tissue (OR = 3.01, 95% CI = 1.05–8.66) (Fig. 2E). Additionally, the NDMT gene signature identified patients that were more likely to be diagnosed with BCLC stage B and C (OR = 2.32, 95% CI = 1.01–5.32) and elevated preoperative AFP (OR = 2.14, 95% CI = 1.27–3.60) (Fig. 2E). These findings suggest that the NDMT subtype consists of an aggressive form of HCC and the NDMT signature may aid in prognosis and help guide treatment and surveillance decisions beyond the current standard.

### Genomic characteristics of the NDMT subtype

**Figure 3:**
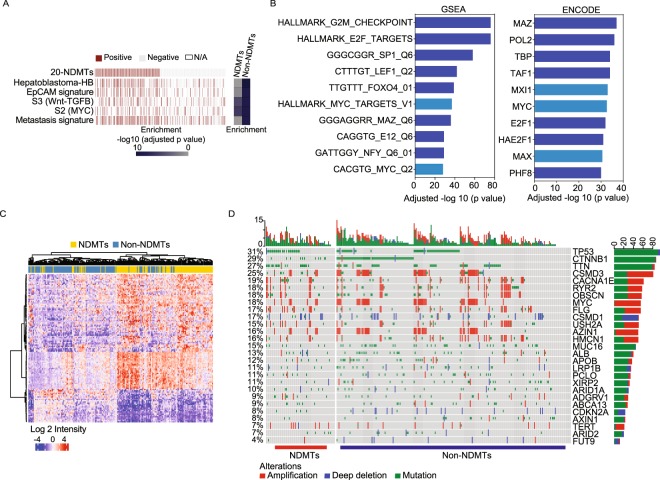
Genomic landscape of NDMTs. (**A**) Comparison of the NDMT tumor subtype to published signatures is indicated in the LCI cohort. Fisher’s exact test was performed to test for enrichment with Bonferonni correction (presented as adjusted p value). (**B**) (Left) Gene set enrichment analysis (GSEA) of differentially expressed genes (DEGs) between NDMTs vs. Non-NDMTs in both the LCI and TCGA-LIHC cohort (p < 0.001). Only genes the overlap of DEGs in both cohorts are shown. (Right) The overlap of DEGs in both HCC cohorts were analyzed using ENCODE to identify up-stream targets (right). Only DEGs in the top ten groups are shown. Light blue represents MYC signaling/targets. (**C**) Heatmap of NELFE/MYC target genes differentially expressed between NDMTs vs. Non-NDMTs (p < 0.001) in the LCI cohort. Student’s t-test was performed to identify DEGs following enrichment analysis for NELFE/MYC target genes. (**D**) Mutation and copy number alterations in the TCGA-LIHC cohort of the most frequently altered genes in HCC. % represents frequency of alterations for the entire cohort. Top graph represents frequency (%) of alterations per sample. Right side graph represents total number of alterations for specified gene.

We next investigated the functional characteristics of the NDMTs with other known HCC subtypes using a nearest template prediction algorithm in the LCI cohort^10,13,17,20–23^. We found that the NDMT subtype consists of HCC with stem-like features as evident by the enrichment of patients with Hepatoblastoma-like features and EpCAM (Fig. 3A). In addition, NDMTs were enriched with patients with metastatic and MYC alterations as identified by Hoshida’s S2 subtype (Fig. 3A)^10^.

To further confirm that NDMTs are enriched with active NELFE/MYC signaling, we performed differential gene expression analyses. First, we identified differentially expressed genes (DEGs) between the NDMT and Non-NDMT subtypes (two-sample t-test, p < 0.001) in the LCI (1,875 DEGs) and TCGA-LIHC cohort (5,281 DEGs). From the two genes lists, we filtered out genes that are up-regulated or down-regulated in both gene lists, resulting in 1,104 DEGs. We then performed gene set enrichment analyses (GSEA) and ENCyclopedia Of DNA Elements (ENCODE) analyses to identify signaling pathways. Consistently, MYC signaling was significantly enriched (Fig. 3B) in GSEA analyses. In addition, ENCODE analyses further confirmed that a significant number of the DEGs between NDMTs and Non-NDMTs were downstream targets of MYC and its partners, MAX and MXI1 (Fig. 3B, right). To confirm that the DEGs between NDMTs and Non-NDMTs were enriched with NELFE/MYC targets, we analyzed DEGs (Benjamin-Hochberg test, false discovery rate of <0.05) in the LCI or TCGA-LIHC cohort separately. We found a significant number of NELFE/MYC targets in the LCI cohort (hypergeometric test, p = 6.5 × 10^−63^) and the TCGA-LIHC cohort (hypergeometric test, p < 0.001) (Fig. 3C, Supplementary Fig. S3A), suggesting that the NDMT HCC subtype consists of active NELFE/MYC signaling.

To investigate the genomic changes between the NDMT and Non-NDMT subtypes, we determined the most frequent somatic mutations and gene copy number alterations for each group, including known genes important for HCC progression (AZIN1, TERT, ARID2, and CDKN2A) in the TCGA-LIHC cohort (Fig. 3D). In the NDMT subtype, we observed TP53 is most frequently mutated at a frequency of 45% as compared to 27% in the Non-NDMT subtype (Supplementary Fig. S3C,D). Consistently, TP53 mutations were more prevalent in the NDMT subtype compared to the Non-NDMT subtype in the LCI cohort (Supplementary Fig. S3B). The NDMT subtype had a CTNNB1 mutation frequency of 16% compared to 32% in the Non-NDMT subtype (Supplementary Fig. S3C,D). Notably, CDKN2A deletions are more prevalent in the Non-NDMT subtype compared to NDMT subtype. The gene signature showed no enrichment for MYC gene amplification for either subtype in both the TCGA-LIHC and LCI cohorts, indicating that the signature identified tumors with active NELFE/MYC signaling and not MYC amplification alone (Fig. 3C, Supplementary Fig. S3B,C).

### Identification of the NDMT subtype in other tumor types

**Figure 4:**
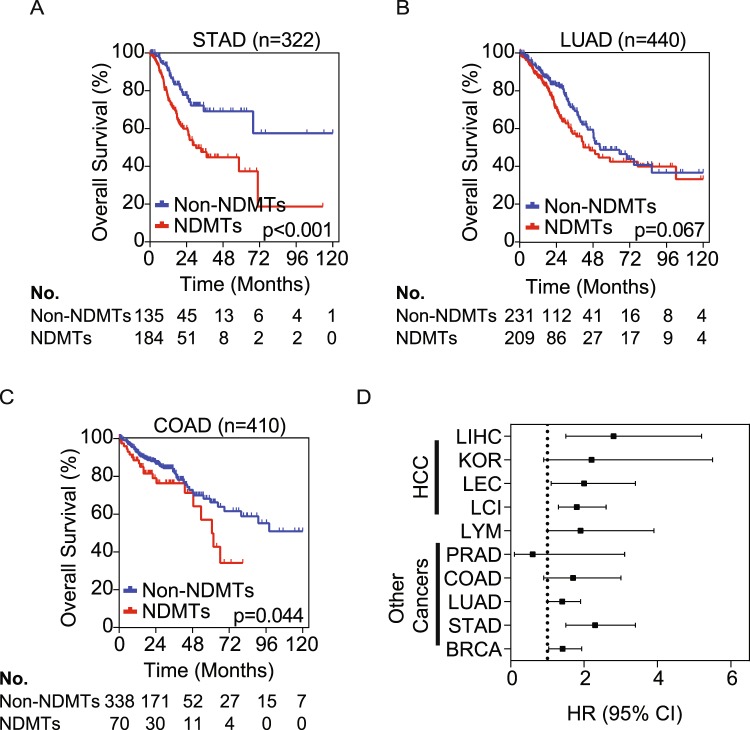
The NDMT gene signature identifies NDMTs in other epithelial tumor types. Kaplan-Meier curve of TCGA datasets (**A**) STAD: stomach adenocarcinoma (**B**) LUAD: lung adenocarcinoma (**C**) COAD: colon adenocarcinoma. P values are from Mantel-Cox log rank test. (**D**) Forest plot of hazard ratios (±95% CI) for OS at 5 years from all cancer types (Liver cancers: LIHC, KOR, LEC, and LCI; other cancers include LYM: Burkitt’s lymphoma, PRAD: prostate adenocarcinoma, BRCA: invasive breast carcinoma).

To assess the gene signature’s ability to predict NDMTs in other cancers, we analyzed data from five commonly occurring solid tumor types using the TCGA database. Datasets used for analyses included stomach adenocarcinoma (STAD), lung adenocarcinoma (LUAD), colon adenocarcinoma (COAD), prostate adenocarcinoma (PRAD), and invasive breast carcinoma (BRCA). In addition, we assessed Burkitt’s lymphoma (LYM) using the dataset from Hummel, *et al*., which consists predominantly of lymphomas with MYC alterations^24^. Accordingly, the gene signature was predictive of OS in the STAD cohort (p < 0.001), COAD cohort (p = 0.044), and with near statistical significance in the LUAD cohort (p = 0.067) (Fig. 4A–C). In the Hummel cohort of 220 lymphoma cases, of which 80% had some form of MYC alteration, the gene signature identified the NDMT subtype with near statistical significance (p = 0.150) (Supplementary Fig. S4A)^24^. Consistent with Hummel *et al*., the NDMT subtype is significantly enriched in lymphoma samples with MYC alterations (Fisher’s exact test, adjusted p < 0.001), whereas the Non-NDMT subtype is significantly enriched with MYC-negative lymphoma samples (Fisher’s exact test, adjusted p = 0.021). Additionally, the gene signature is more robustly associated with a poor survival in HCC than other tumor types (Fig. 4D). Interestingly, in prostate and breast cancer, the gene signature did not classify tumors into the NDMT or Non-NDMT subtypes with statistical significance (Supplementary Fig. S4A,B). This is consistent with previous work as the most common alterations in prostate cancer are androgen-regulated promoter fusions with members of the E26 transformation-specific transcription factors. Furthermore, MYC and NELFE alterations are found in only 8% and 0.8% of prostate cancer patient samples, respectively^25^.

## Discussion

The heterogeneous nature of cancer is related to the diverse etiologies associated with the individual disease, which is exemplified in HCC. In recent years, the discovery of targeted therapy has led to novel agents that act directly on signaling cascades important for tumor survival. Although some therapies have provided modest improvements, recent failures in the testing of systemic drugs for advanced cancers suggest a need for better patient stratification and novel therapies. In the current study, we developed a gene signature that could serve as a predictive marker of survival for HCC with an NDMT biology. Accordingly, we validated this signature through a range of racial/ethnic backgrounds and underlying etiologies. Varying etiologies of chronic liver disease contribute to different signaling pathways that drive tumorigenesis and promote tumor heterogeneity, making treatment difficult. However, our gene signature consistently predicted OS, indicating that the signature is a strong predictor of tumors with a NELFE/MYC biology associated with poor outcome. Since the signature consists of oncogenic MYC target genes regulated by NELFE, it is reasonable that the NDMT subtype consists of tumors that are stem-like, have dysregulated WNT signaling, and metastatic features^5,26^.

The systemic and locoregional therapies offer only marginal improvements in survival, liver transplantation and hepatic resection remain the gold standard for early HCC. However, the selection for curative therapy is based on physical features of the tumor, failing to recognize the underlying biology of the tumor^27^. In addition, there are currently many staging systems for HCC, most notably the BCLC and the AJCC/TNM 8^th^ edition^28^. While TNM staging relies on accurate histopathologic diagnosis of both the tumor and local lymphatics, BCLC stratifies patients based on liver function followed by the extent of disease. Recent studies have shown a lack of prognostic accuracy between pT3 and pT4 tumors, thus limiting TMN for late stage tumors. In addition, when tumors are staged using the BCLC classification, the subjective component of performance status and Child-Pugh criteria leads to selection bias, where the majority of patients are classified as BCLC stage B, making this an imperfect system^28,29^. Moreover, biomarkers such as AFP have been utilized to predict survival outcome, however, the results remain inconsistent. Recent work by Berry *et al*. implicate AFP levels > 320 ng/ml as an independent predictor of recurrence after transplantation^30^. Conversely, Farinati *et al*. found that 43% of patients with early HCC had normal AFP levels^12^. While AFP may be strongly correlated with advanced disease, there are clear shortcomings in early disease. Thus, the ability to identify tumors based on their biology, independent of AFP or BCLC staging, or in combination with BCLC staging, solidifies the NDMT as a prognostic tool with possible therapeutic value.

Multiple HCC gene signatures have been developed for translational application, including multiple proliferation signatures, an early metastatic gene signature, a high risk cirrhotic signature, an inflammation/immune response-related signature, and stem-like gene signatures; most of which are associated with poor outcome^10,18,19,31–36^. While these signatures have potential for prognostication in HCC, none have been introduced into clinical practice. As evident in our analyses, all six gene signatures performed variably across different HCC cohorts. This is mostly due to the vast inter- and intra-tumor heterogeneity of HCC and the underlying etiologies associated with the tumor, including HBV/HCV, alcoholic- and non-alcoholic fatty liver disease. Moreover, the patients tested are predominantly Asians or Caucasians with at most three dominant etiologies, including HBV, HCV, or alcohol^18^. Most importantly, some signatures are predominantly developed using genes that are tumor-specific but not functionally inter-related and thus, ignores the tumor biology that drives HCC. The NDMT signature in contrast performed consistently across the three cohorts tested, suggesting that utilizing genes that are functionally important, such as the NELFE/MYC signaling pathway, may be better at identifying more homogeneous subtypes.

MYC alteration is found in more than 30% of HCC and its signaling can be altered without concomitant gene amplification or mutation^3–5^. Instead, its expression can be altered through upstream signal transduction, epigenetic changes and the regulation of mRNA or protein stability^4,7^. This is evident in HCC that are driven by NELFE, an RNA binding protein that can interact with MYC-associated mRNA targets or the MYC protein to directly promote MYC-induced tumorigenesis^5^. Moreover, NELFE is upregulated in 10–17% of HCC, whereas the dysregulation of any combination of NELFE, MYC, or NELFE/MYC makes up ~38% of HCC^5^. Although our gene signature did not identify MYC amplified tumors, given the extent to which both genes play an important role in HCC progression together or independently, the NDMT gene signature could serve as a predictive marker of survival for not only tumors driven by NELFE/MYC, but also MYC or NELFE driven tumors.

In conclusion, the NDMT gene signature is robust in identifying the NDMT subtype with the ability to add valuable prognostic information in HCC. Moreover, we demonstrated that the utility of the NDMT signature is not limited to HCC. MYC alteration is found in 21% of all TCGA samples across 33 different tumor types and NELFE is upregulated in most solid tumors^4^. When we tested our signature among five common cancers, we found that our gene signature can identify NDMTs in three of the six cancer types, including STAD, LUAD and COAD. These results suggest the signature identifies a functionally specific tumor type whose driver is NELFE/MYC signaling. Furthermore, the signature is independent of previously established diagnostic tools such as TNM, BCLC and AFP. Future work will be needed to demonstrate the full breadth of the signature and its role in shaping therapeutic decisions. However, the NDMT would provide prognostic value across etiologies without complete pathologic staging and eliminate clinician bias, which may prove to be a valuable adjunct to the current staging systems.

## Methods

### Patient cohorts

Each HCC dataset is publicly available at the Gene Expression Omnibus (GEO; http://www.ncbi.nlm.nih.gov/geo) or the TCGA (https://cancergenome.nih.gov/). The data for the Liver Cancer Institute (LCI) cohort (GSE14520), Laboratory of Experimental Carcinogenesis (LEC) (GSE1898 and GSE4024), and TCGA-LIHC are previously described^10,13,14^. For the Korean cohort data, we merged two independent datasets together to improve our prediction due to the low number of samples in each dataset. Accordingly, 88 HCC cases are from Keimyung University and Korea University and 90 cases are from Seoul National University Hospital (GSE15765)^15,16^.

The TCGA datasets (RNASeq and clinical tables) for gastric (STAD), lung (LUAD), colon (COAD), prostate (PRAD) and breast (BRCA) were downloaded (03-27-2015) using the R (v3.12) TCGA Assembler package, http://www.compgenome.org/TCGA-Assembler)^37^. For the lymphoma cohort (LYM), data was downloaded through the Oncomine Research Edition database (https://www.oncomine.org/resource/login.html)^24,38^. To curate the TCGA-LIHC for BCLC staging, we used ECOG performance, tumor size, number and multi-nodularity, as well as Child Pugh score. All cases with incomplete data were not included.

### Development of NDMT prognostic index

We previously identified 68 MYC associated genes that were also regulated by NELFE^5^. From the 68-gene list, we identified 20 genes that had at least a 1.5-fold change between tumor and non-tumor in HCC samples found in the LCI cohort. Log 2 expression values from the LCI cohort were transformed into z-score values followed by Survival Risk Prediction analysis (BRB Array Tools v.4.5.1)^11^. Using the 20 genes, survival risk prediction analysis was performed in the training LCI Cohort. Briefly, the algorithm applies univariate Cox proportional hazards regression following principal components analysis, which transforms possible correlated variables into two principal components. The result of this algorithm are regression coefficients (weight) related to survival data for each gene in the training dataset. Next, the prognostic index score is calculated using the weighted average of the principal component values from the Cox regression from the first step. Lastly, patients are then partitioned into two risk (high = NDMT, low = Non-NDMT) groups of equal size using the median as a cutoff. Kaplan–Meier curve analysis, 10-fold cross validation and 1000 permutations based on log rank statistic are performed to evaluate the accuracy of the score system. Because we wanted to ensure all 20 genes were used, the significance threshold of the Cox model was set to 0.999. For cross validation, the index score was calculated by summing the product of the expression level of a gene and its corresponding regression coefficient to determine if a new sample should be classified as NDMT (worse overall survival) or Non-NDMT tumor types (better OS). The prognostic index can be computed by the formula:

$$PI=\sum _{i}{w}_{i}{x}_{i}-0.620546$$

where *w*_*i*_ and *x*_*i*_ are the weight and logged gene expression for the *i*-th gene. The gene list and weight for each gene can be found in Supplementary Fig. S1. A new sample is predicted as NDMT (Non-NDMT) if its prognostic index (PI) is larger than (smaller than or equal to) −0.062139.

For all validation analyses, the log 2 expression values for each gene were transformed into z-score values, with the exception for the Burkitt’s lymphoma cohort, which was downloaded from Oncomine (https://www.oncomine.org/resource/login.html) in z-score format. Level 3 RNASeq data from the TCGA were log 2 (log 2 + 1) transformed followed by z-score transformation using R (version 3.3.3). OS or recurrence was then analyzed using Kaplan-Meier and Cox regression analyses.

### Statistical Analysis

In all statistical analyses for this study, a two-sided p-value of <0.05 is considered statistically significant. Clinical data was evaluated using Chi-squared test. For enrichment analysis, Fisher’s exact test was performed followed by Bonferroni correction to adjust for multiple hypothesis testing. Patient survival outcome was evaluated by using Kaplan-Meier analysis with Cox-Mantel log-rank test to determine statistical significance. Statistics were calculated using GraphPad Prism 7.0 (GraphPad, San Diego, CA). For heatmaps, we performed hierarchal clustering analyses with Pearson correlation Ward linkage.

Univariate and multivariate analyses was performed with Cox proportional hazards regression analysis using STATA 14.0 (College Station, TX). The association between each clinical variable and survival outcome was first evaluated with univariate analysis followed by multivariate analysis, which included clinical variables with a p-value < 0.05 in the univariate analysis. Tumor size was not used in multivariate analyses because it was already used to determine tumor stage. No multi-collinearity of covariates was found, and the proportional hazards assumption was met in the final models.

Nearest Template Prediction (NTP) was used for gene signature determination (not including the NDMT signature) across all HCC cohorts, we used Nearest Template Prediction algorithm^23^ with R package CMScaller^39^. In short, a set of genes is used as a template to define different classes of biological samples, then the samples are categorized based on the provided gene set, and finally prediction confidence is calculated based on resampling technique. The liver cancer signatures used for the comparison were Hepatoblastoma signature^13^, EpCAM signature^20^, Wnt-TGFB and MYC signatures^21^ and Metastasis signature^10^. All gene expression values from each cohort were log2- and z-transformed before NTP analyses.

Time-dependent ROC calculation: Performance of multiple gene signatures based on gene expression from tumor cells were compared by using two-year time-dependent receiver operating characteristics (ROC) from censored data (Heagarty *et al*., 2000) with R package survivalROC (https://cran.r-project.org/web/packages/survivalROC/index.html). The time-dependent ROC algorithm used in this study is cumulative sensitivity, which calculates probability of a patient that will have a certain outcome (in this case the outcome is death) before a specific time, and dynamic specificity, which calculate the probability that a patient has a certain marker value (such as risk score or class status) less than or equal to a certain threshold after a certain time. In this study we chose cumulative sensitivity and dynamic specificity because we specifically defined two different time points for evaluation as two- and five-year periods. The survival estimator used in this calculation is Kaplan-Meier estimator. For the NDMT signature, the marker used in the time-dependent ROC was the prognostic index values. For all other signatures, the marker used was the distance measures from the signature of interest. 95% confidence interval calculation was performed by using 1,000 iteration of ordinary bootstrap method with R package.

Heatmaps of NDMT vs Non-NDMT from the LCI and TCGA-LIHC cohorts were generated by ComplexHeatmap R package. The genes presented in the heatmaps represent differentially expressed genes (Student’s test with FDR via Benjamini-Hochberg, adjusted p-value p < 0.00001) between NDMT vs Non-NDMTs and fold-change >1.4 or <0.714.

## Supplementary information

Supplementary Figures

## Data Availability

The datasets generated during or analyzed during the current study are available from the corresponding authors upon reasonable request.
