# Supplementary material for: NELFE-Dependent MYC Signature Identifies a Unique Cancer Subtype in Hepatocellular Carcinoma
Source: Sci Rep. 2019 Mar 4;9:3369. doi: 10.1038/s41598-019-39727-9 (PMC6399236; doi:10.1038/s41598-019-39727-9)
Supplement: Supplementary file 1 — Supplementary Figures [file 41598_2019_39727_MOESM1_ESM.pdf]

NELFE-Dependent MYC Signature Identifies a Unique Cancer Subtype in Hepatocellular Carcinoma

Hien Dang[1,4*], Yotsawat Pomyen[1,3], Sean P. Martin[1], Dana A. Dominguez[1], Sun Young Yim[2], Ju-Seog Lee[2], Anuradha Budhu[1], Ashesh A. Shah[5], Adam S. Bodzin[5], and Xin Wei Wang[1*]

[1]Laboratory of Human Carcinogenesis, Center for Cancer Research, National Cancer Institute, Bethesda, Maryland, United States

[2]Department of Systems Biology, Division of Cancer Medicine, UT MDACC, Houston, TX, United States

[3]Translational Research Unit, Chulabhorn Research Institute, Bangkok, 10210, Thailand

[4]Department of Surgery, Division of Surgical Research, Thomas Jefferson University, Philadelphia, PA, United States

[5]Department of Surgery, Division of Transplantation, Thomas Jefferson University, Philadelphia, PA, United States

*Co-corresponding authors

Xin Wei Wang: xw3u@nih.gov

Hien Dang: hien.dang@jefferson.edu

Supplemental Table 1. Demographic, clinical and laboratory characteristics of HCC patients in the LCI, TCGA-LIHC, and LEC cohorts.

Cohort	LCI			TCGA-LIHC			LEC			KOREAN		
**Clinical variable**	**Non-NDMTs (121)**	**NDMTs (120)**	**p value[a]**	**Non-NDMTs (273)**	**NDMTs (91)**	**p value[a]**	**Non-NDMTs (107)**	**NDMTs (32)**	**p value[a]**	**Non-NDMTs (115)**	**NDMTs (43)**	**p value[a]**
**Demographics**												
**Sex**			0.348			**0.009**			0.489			0.518
Male	106 (87%)	102 (85%)		190 (70%)	48 (53%)		77 (72%)	25 (78%)		96 (83%)	34 (79%)	
Female	13 (11%)	18 (15%)		75 (27%)	37 (41%)		30 (28%)	7 (22%)		19 (17%)	9 (21%)	
No. (%) missing data	2 (2%)	0 (0%)		8 (3%)	6 (6%)		0 (0%)	0 (0%)		0 (0%)	0 (0%)	
**Age**			0.841			0.758			**0.008**			0.862
≥50 yr	53 (44%)	55 (46%)		219 (80%)	69 (76%)		77 (72%)	15 (47%)		84 (73%)	32 (74%)	
<50 yr	66 (54%)	65 (54%)		46 (17%)	13 (14%)		30 (28%)	17 (53%)		31 (27%)	11 (26%)	
No. (%) missing data	3 (2%)	0 (0%)		8 (3%)	9 (10%)		0 (0%)	0 (0%)		0 (0%)	0 (0%)	
**Risk factors**												
**Cirrhosis**			**0.03**			0.092			0.653			0.134
Yes	105 (87%)	115 (96%)		99 (36%	9 (25%)		52 (49%)	17 (53%)		40 (35%)	2 (5%)	
No	14 (11%)	5 (4%)		66 (24%)	27 (75%)		55 (51%)	15 (47%)		46 40%)	0 (0%)	
No. (%) missing data	2 (2%)	0 (0%)		108 (40%)	0 (0%)		0 (0%)	0 (0%)		29 (25%)	41 (0%)	
**BMI**			0.824			0.340			N/A			N/A
Under weight, BMI <18.5	6 (5%)	4 (3%)		18 (7%)	2 (2%)							
Overweight/Obesity, BMI >24	49 (40%)	38 (32%)		140 (51%)	32 (35%)							
No. (%) missing data	66 (55%)	48 (65%)		115 (42%)	57 (63%)							
**HBV status**			**0.008**			N/A			N/A			N/A
CC	93 (77%)	76 (63%)										
AVR-CC	22 (18%)	40 (33%)										
No. (%) missing data	6 (5%)	4 (4%)										
**Liver function factors**												
**AFP**			**0.011**			**p<0.001**			0.200			0.169
Normal, ≤400 ng/ml	72 (60%)	53 (44%)		169 (62%)	32 (35%)		60 (56%)	13 (40%)		89 (77%)	29 (67%)	
Abnormal, >400 ng/ml	45 (37%)	65 (54%)		40 (15%)	23 (25%)		40 (37%)	15 (47%)		25 (22%)	14 (33%)	
No. (%) missing data	4 (3%)	2 (2%)		64 (23%)	36 (40%)		7 (7%)	4 (13%)		1 (1%)	0 (0%)	
**ALT**			0.165			N/A			N/A			**0.020**
ALT < 50	75 (62%)	65 (54%)								89 (77%)	22 (51%)	
ALT > 50	44 (36%)	55 (46%)								26 (23%)	16 (37%)	
No. (%) missing data	2 (2%)	0 (0%)								0 (0%)	5 (12%)	
**Tumor features**												
**Histological grade**			0.040			**0.071**			**0.442**			N/A
I	6 (5%)	5 (4%)		42 (15%)	7 (8%)		2 (2%)	0 (0%)				
II-IV	52 (43%)	51 (43%)		207 (76%)	74 (81%)		101 (94%)	30 (94%)				
No. (%) missing data	63 (52%)	64 (53%)		24 (9%)	10 (11%)		4 (4%)	2 (6%)				
**TNM staging**			**0.034**			**p<0.001**			0.503			**0.003**
I	58 (48%)	36 (30%)		147 (54%)	27 (32%)		4 (4%)	0 (0%)		82 (71%)	19 (44%)	
II+III	37 (31%)	44 (37%)		115 (42%)	57 (68%)		62 (58%)	7 (22%)		33 (29%)	23 (54%)	
No. (%) missing data	26 (21%)	40 (33%)		11 (4%)	49 (54%)		41 (38%)	25 (78%)		0 (0%)	1 (2%)	
**Microvascular invasion**			**0.019**			**0.021**			0.110			**<0.001**
No	83 (68%)	66 (55%)		160 (59%)	34 (37%)		21 (20%)	1 (3%)		98 (85%)	23 (53%)	
Yes	36 (30%)	54 (45%)		73 (27%)	30 (33%)		43 (40%)	10 (31%)		16 (14%)	20 (47%)	
No. (%) missing data	2 (2%)	0 (0%)		40 (15%)	27 (30%)		43 (40%)	21 (66%)		1 (1%)	0 (0%)	
**Child-Pugh class**			0.727			0.659			N/A			0.878
A	39 (32%)	40 (33%)		166 (61%)	39 (43%)					85 (74%)	2 (5%)	
B	18 (15%)	16 (14%)		17 (6%)	3 (3%)					1 (1%)	0 (0%)	
No. (%) missing data	64 (53%)	64 (53%)		90 (33%)	49 (54%)					29 (25%)	41 (95%)	
**Tumor size**			0.517			N/A			**0.039**			0.325
>3 cm	39 (32%)	35 (26%)					83 (76%)	30 (94%)		33 (29%)	9 (21%)	
≤3 cm	79 (65%)	85 (71%)					24 (22%)	2 (6%)		82 (71%)	34 (79%)	
No. (%) missing data	3 (3%)	0 (0%)					0 (0%)	0 (0%)		0 (0%)	0 (0%)	
**Multinodular tumor**			0.093			N/A			N/A			0.748
No	98 (82%)	88 (73%)								68 (59%)	1 (2%)	
Yes	21 (18%)	32 (27%)								7 (6%)	0 (0%)	
No. (%) missing data	3 (3%)	0 (0%)								40 (35%)	42 (98%)	
**BCLC staging**			**0.008**			0.371			NA			0.658
0&A	93 (76%)	77 (64%)		99 (37%)	0 (0%)					56 (49%)	1 (2%)	
B&C	18 (15%)	35 (29%)		102 (37%)	30 (33%)					30 (26%)	1 (2%)	
No. (%) missing data	10 (9%)	8 (7%)		72 (26%)	61 (67%)					29 (25%)	41 (96%)	
**Recurrent Metastasis**			0.827			N/A			N/A			NA
No	76 (63%)	75 (63%)										
Yes	43 (35%)	45 (37%)										
No. (%) missing data	2 (2%)	0 (0%)										

Abbreviations: NA: Not available, HBV-hepatitis B virus, AVR-CC, active viral replication chronic carrier, CC, chronic carrier, HCV-hepatitis C virus, AFP-alpha-fetoprotein, ALT-alminotransferase, BCLC-Barcelona-Clinic liver cancer, and BMI-body mass index

[a]Chi-squared test

Supplemental Figure S1

A

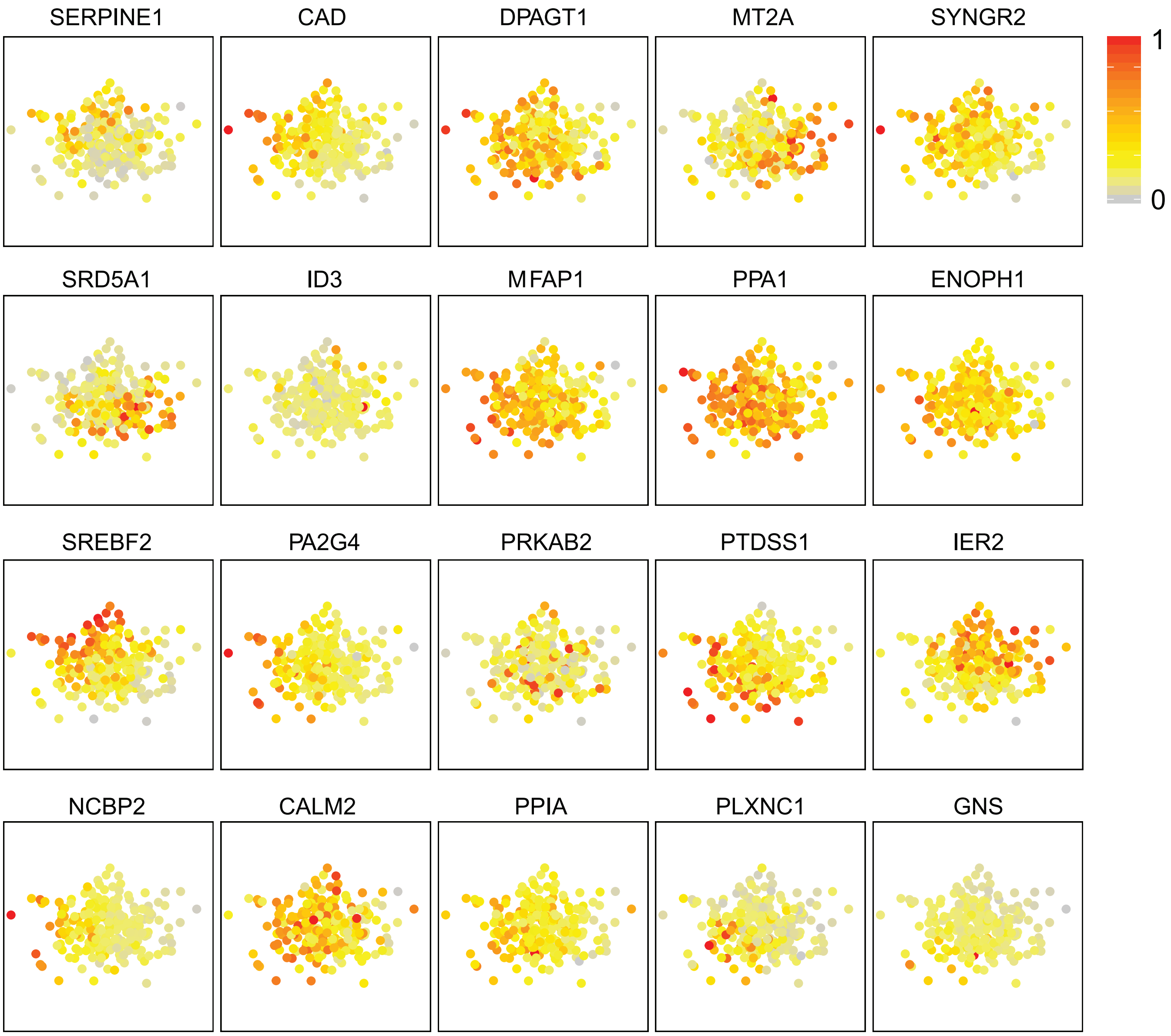

B

Gene Id	Weights (w_i)
CAD	0.061746
CALM2	0.042413
DPAGT1	0.020167
ENOPH1	0.048531
GNS	0.031138
ID3	-0.019005
IER2	0.049337
MFAP1	0.022254
MT2A	-0.152551
NCBP2	0.092179
PA2G4	0.03582
PLXNC1	-0.015257
PPA1	0.011134
PPIA	0.0353
PRKAB2	-0.032401
PTDSS1	0.040496
SERPINE1	0.608612
SRD5A1	-0.19421
SREBF2	0.097164
SYNGR2	0.078953

Supplementary Figure S1. NELFE dependent MYC target (NDMTs) genes. (A) Gene expression of 20-NDMTs in the LCI cohort. (B) Regression coefficient (Weight) of each gene in the 20-NDMT signature used to calculate the index for each patient.

Supplemental Figure S2

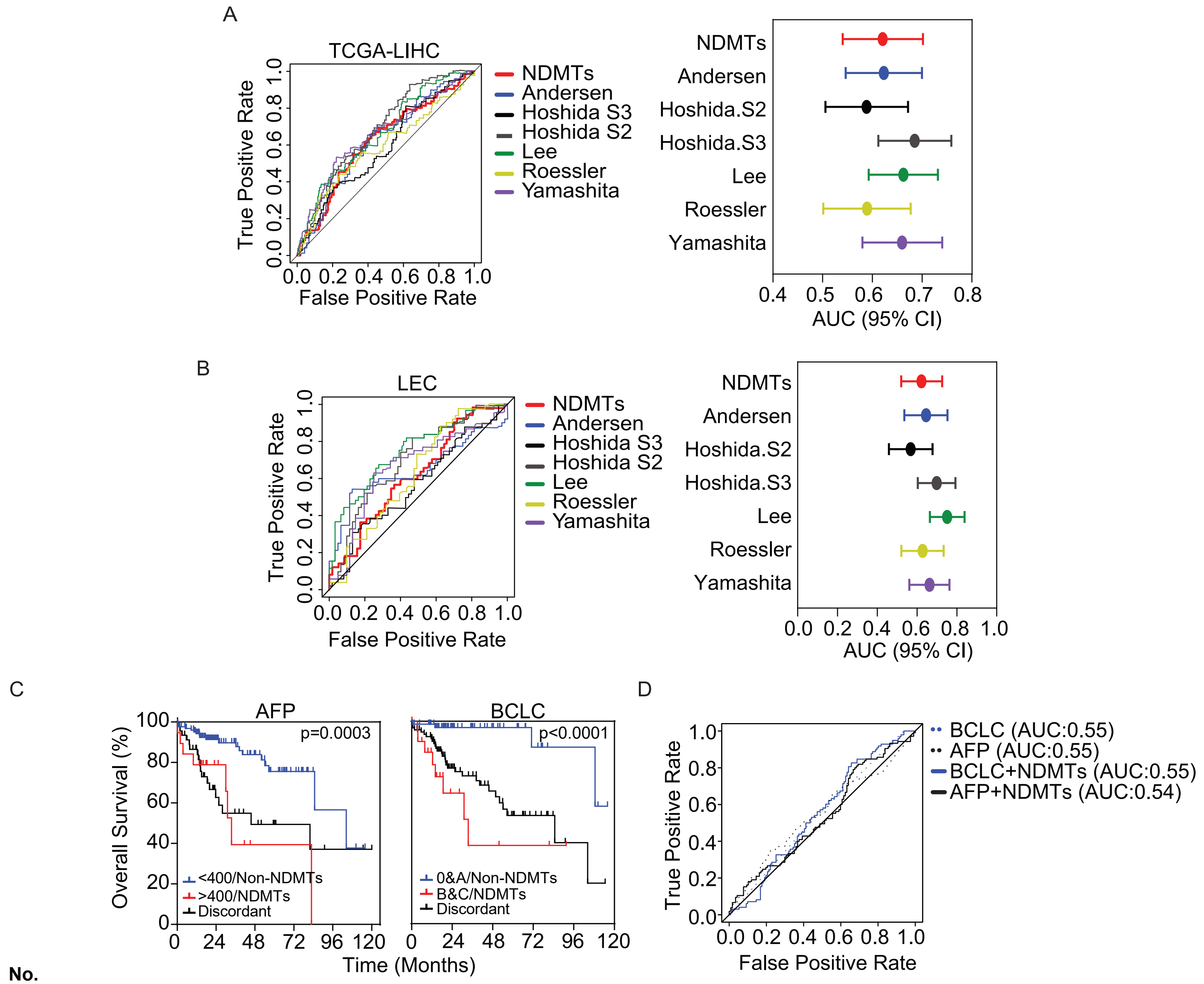

**No.**												
<400/Non-NDMTs	128	64	38	15	4	1	72	36	22	10	4	1
>400/NDMTs	19	8	2	2	1	1	21	7	4	3	1	1
Discordant	47	17	11	5	3	1	45	45	10	10	3	1

Supplementary Figure S2. NDMT performance in HCC cohorts. ROC curves for the (A) TCGA-LIHC and (B) LEC cohorts comparing the NDMT gene signature with Non-NDMTs. Forest plot represents AUC (±95% Confidence Interval). (C) Kaplan-Meier curve survival analyses of the LCI cohort stratified by AFP and BCLC staging with NDMTs or Non-NDMTs. P value is from Mantel-Cox log rank analyses. (D) Time-dependent ROC curve analyses of the TCGA-LIHC cohort at two years of Bacrecelon-Clinic Liver Cancer (BCLC), alpha-feto protein (AFP), BCLC +NDMTs, or AFP+NDMTs with associated area under the curve values (AUC).

Supplemental Figure S3

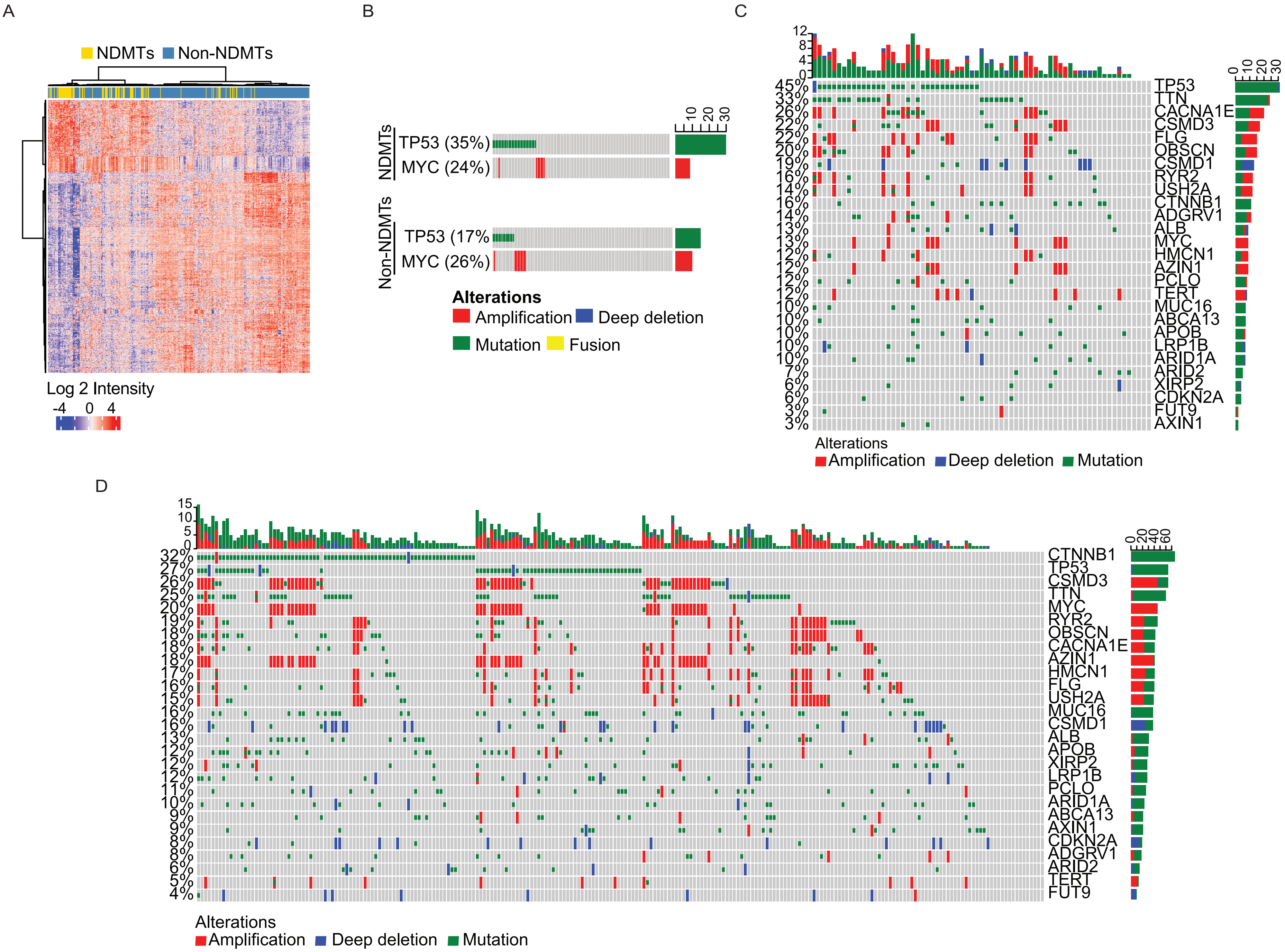

Supplementary Figure S3. Genomic alterations of NDMTs. (A) Heatmap of NELFE/MYC target genes differentially expressed between NDMTs and Non-NDMTs ($p<0.001$) in TCGA-LIHC cohort. Student's t-test was performed to identify DEGs following enrichment analysis for NELFE/MYC target genes. (B) TP53 mutation and MYC gene copy number alteration analyses in the LCI cohort. Mutation and gene copy number alterations of most frequently altered genes in NDMTs (C) and Non-NDMTs (D) TCGA-LIHC cohort.

Supplemental Figure S4

A

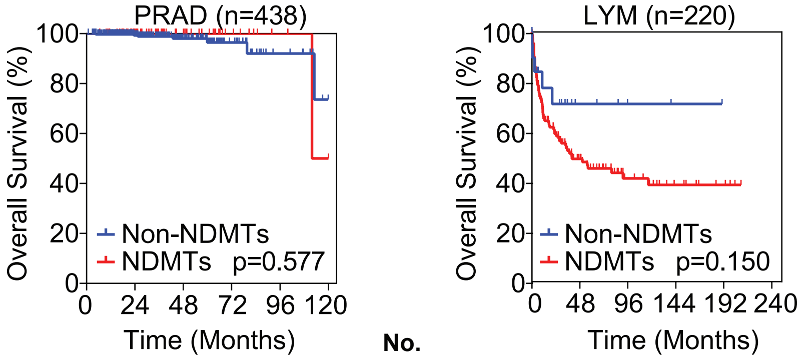

No.						
Non-NDMTs	364	241	91	31	11	2
NDMTs	72	56	25	11	4	1

No.						
Non-NDMTs	21	6	3	2	1	1
NDMTs	134	42	19	10	5	1

B

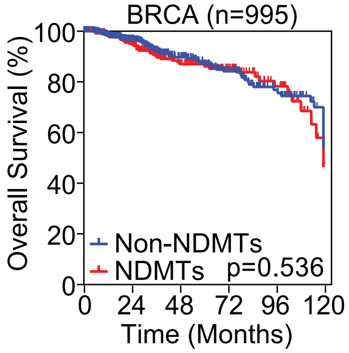

No.						
Non-NDMTs	610	340	182	114	64	30
NDMTs	383	223	126	76	37	10

Supplementary Figure S4. The NDMT subtype in other tumor types. The NDMT gene signature identifies NDMTs in Burkitt's lymphoma (LYM) but not in prostate adenocarcinoma (PRAD). (B) Kaplan-Meier curve of TCGA breast cancer (BRCA) cohort.
